# Supplementary material for: Thermal adaptation best explains Bergmann’s and Allen’s Rules across ecologically diverse shorebirds
Source: Nat Commun. 2022 Aug 11;13:4727. doi: 10.1038/s41467-022-32108-3 (PMC9372053; doi:10.1038/s41467-022-32108-3)
Supplement: Supplementary file 1 — Supplementary Information [file 41467_2022_32108_MOESM1_ESM.pdf]

SUPPLEMENTARY INFORMATION

McQueen et al.

Thermal adaptation best explains Bergmann's and Allen's Rules across ecologically diverse shorebirds

Table S1. List of shorebird species included in the study.

Common name	Scientific name
Curlew Sandpiper	*Calidris ferruginea*
Sharp-tailed Sandpiper	*Calidris acuminata*
Red-necked Stint	*Calidris ruficollis*
Red Knot	*Calidris canutus*
Great Knot	*Calidris tenuirostris*
Sanderling	*Calidris alba*
Bar-tailed Godwit	*Limosa lapponica*
Far Eastern Curlew	*Numenius madagascariensis*
Whimbrel	*Numenius phaeopus*
Ruddy Turnstone	*Arenaria interpres*
Common Greenshank	*Tringa nebularia*
Terek Sandpiper	*Xenus cinereus*
Grey-tailed Tattler	*Heteroscelus brevipes*
Pacific Golden Plover	*Pluvialis fulva*
Grey Plover	*Pluvialis squatarola*
Lesser Sand Plover	*Charadrius mongolus*
Greater Sand Plover	*Charadrius leschenaultii*
Red-kneed Dotterel	*Erythrogonys cinctus*
Red-capped Plover	*Charadrius ruficapillus*
Masked Lapwing	*Vanellus miles*
Common Tern	*Sterna hirundo*
Whiskered Tern	*Chlidonias hybrida*
Little Tern	*Sterna albifrons*
Caspian Tern	*Sterna caspia*
Great Crested Tern	*Sterna bergii*
Black-winged Stilt	*Himantopus himantopus*
Banded Stilt	*Cladorhynchus leucocephalus*
Red-necked Avocet	*Recurvirostra novaehollandiae*
Pied Oystercatcher	*Haematopus longirostris*
Sooty Oystercatcher	*Haematopus fuliginosus*

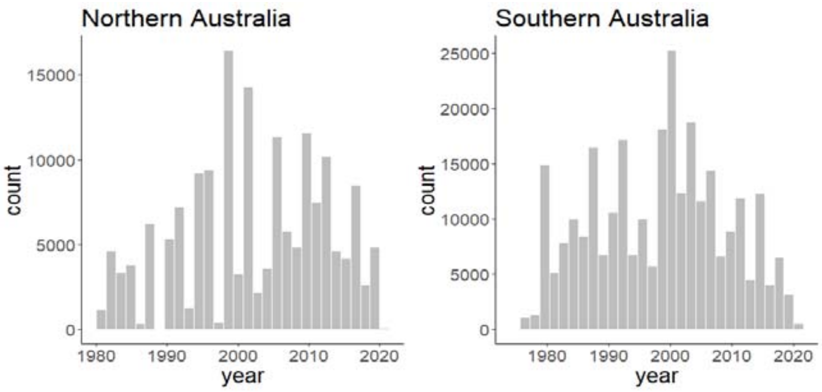

Figure S1. Sampling effort in northern Australia and southern Australia over time, showing samples collected are roughly consistent across years at the two locations.

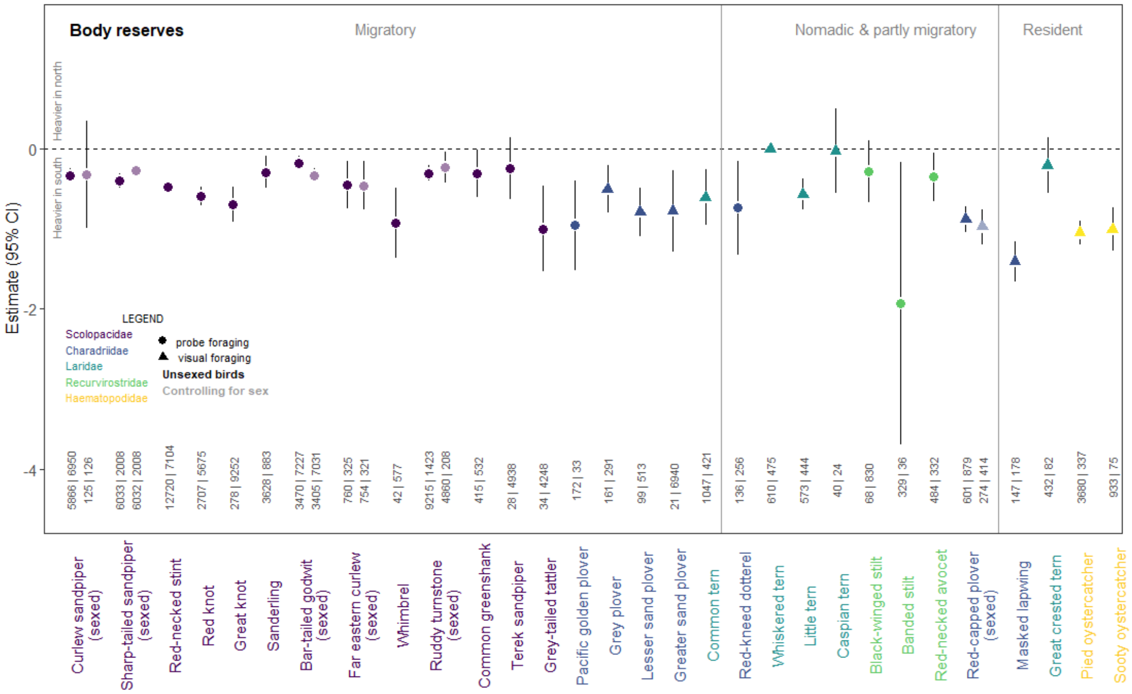

Figure S2. Estimates of energetic resources (assessed as differences in log-transformed mass while controlling for wing length) in northern populations relative to southern populations of 30 shorebird species. Points above the line indicate greater energetic resources in northern populations. Log-transformed mass was scaled and centred, so effect sizes are comparable across species. Points represent the estimates for effect of location derived from linear mixed models. Error bars show 95% confidence intervals. Effects are shown for species with different migration behaviours (migratory, nomadic/partially migratory, and residential) and foraging methods (probe and visual). Estimates controlling for sex differences in morphology are shown for species where individuals could be reliably sexed. Sample sizes are shown for birds caught at different locations (southern observations | northern observations).

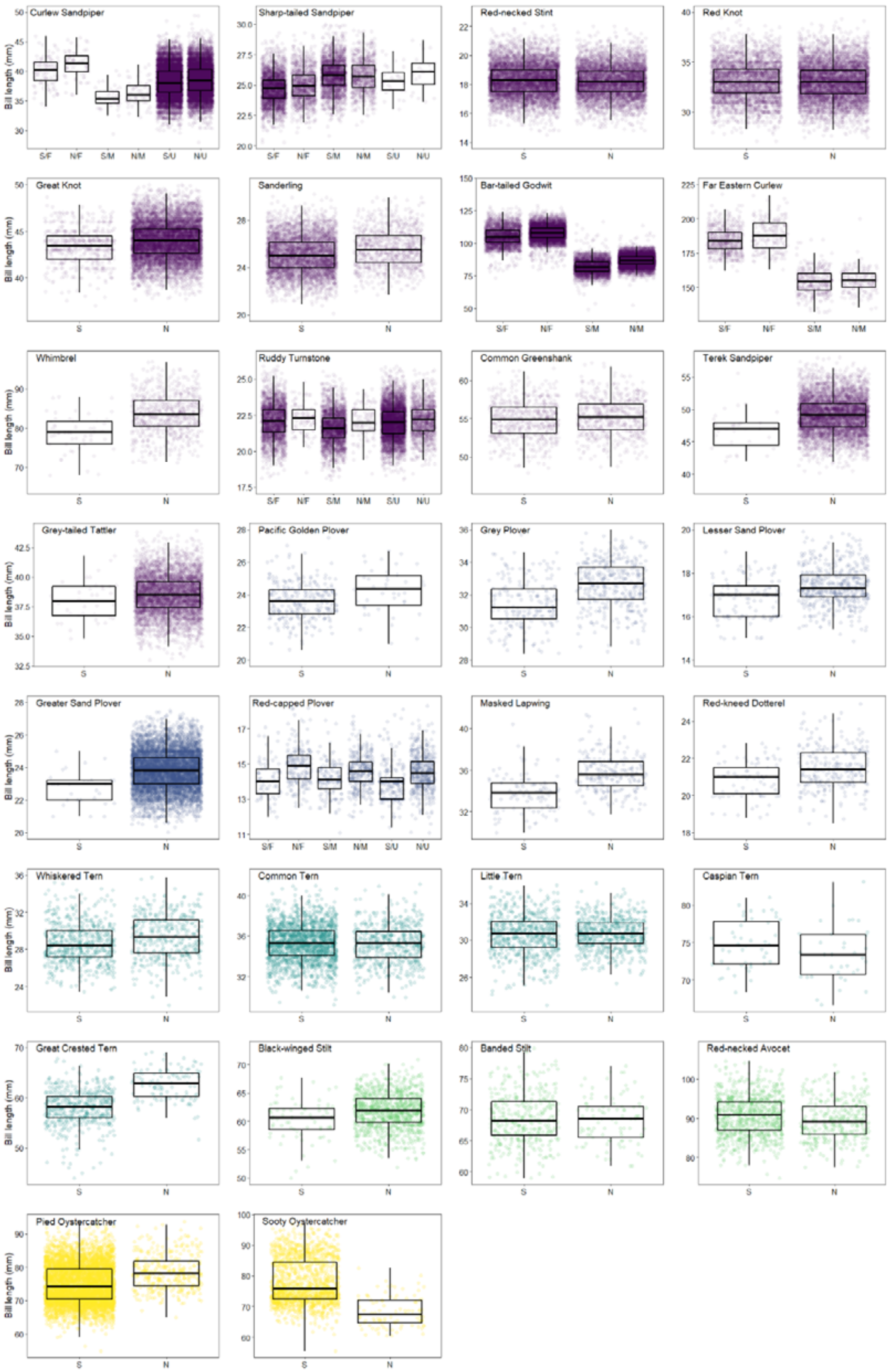

Figure S3. Boxplots showing median (centre), interquartile range (box), and maxima/minima minus outliers (whiskers) of bill length (mm) for shorebird populations in southern (S) and northern (N) Australia. Data are shown for 30 species. Where possible, data are shown separately according to sexes (F = female, M = male, U = unknown). Colours show representatives of different families (as indicated in Figure S1).

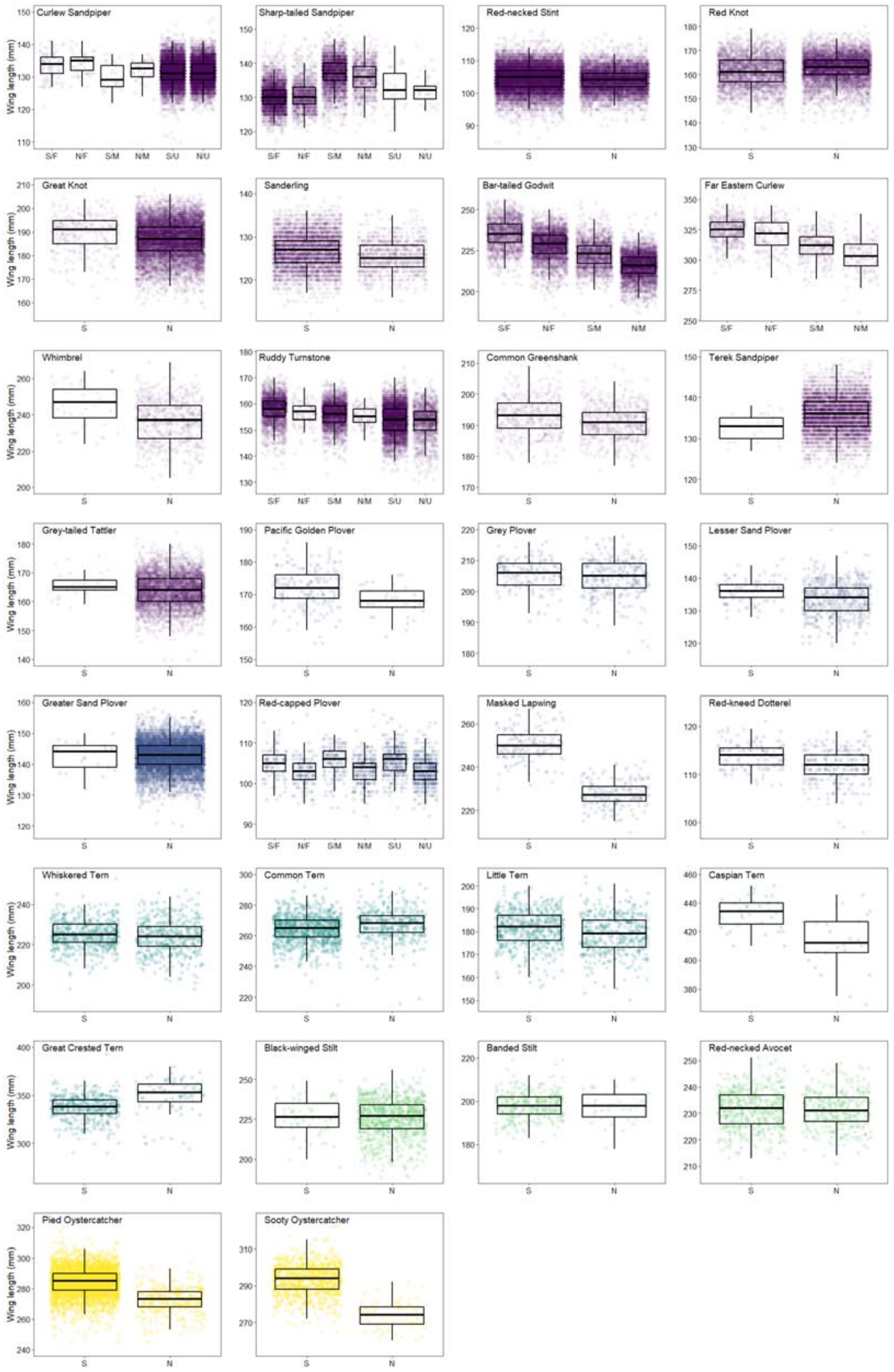

Figure S4. Boxplots showing median (centre), interquartile range (box) and maxima/minima minus outliers (whiskers) of wing length (mm) for shorebird populations in southern (S) and northern (N) Australia. Data are shown for 30 species. Where possible, data are shown separately according to sexes (F = female, M = male, U = unknown). Colours show representatives of different families (as indicated in Figure S1).

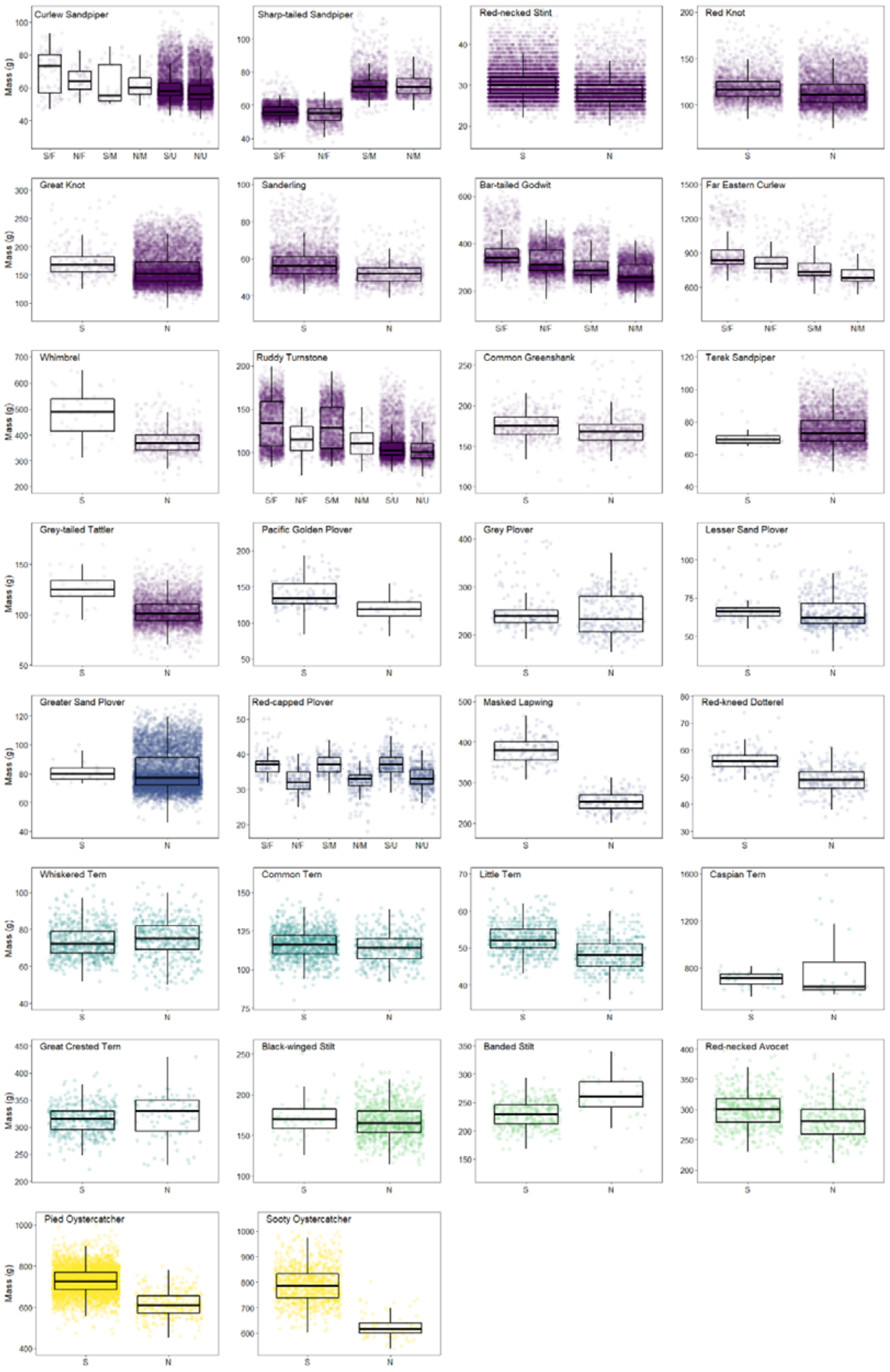

Figure S5. Boxplots showing median (centre), interquartile range (box) and maxima/minima minus outliers (whiskers) of mass (g) for shorebird populations in southern (S) and northern (N) Australia. Data are shown for 30 species. Where possible, data are shown separately according to sexes (F = female, M = male, U = unknown). Colours show representatives of different families (as indicated in Figure S1).

Table S2. Phylogenetic generalised linear mixed model output for predictors of relative bill length of Australian shorebirds. Bill length was log-10 transformed to improve normality; differences in bill length are shown controlling for wing length (as an indicator of body size). Effects are expressed relative to adult, migratory shorebirds in southern Australia, which use their bills to probe for prey, and were sampled during summer-spring or the wet season. Sample size is N = 99,443 observations from 30 shorebird species. Three model outputs show the (i) model without interactions, referenced in the main results, and models testing for an interaction between (ii) location and migration behaviour and (iii) location and foraging method.

**Model showing main results**			
**Random effects**	**Variance**		
Species	0.013		
Phylogeny	0.004		
Residual	0.001		
**Fixed effects**	**Estimate**	**Lower CI**	**Upper CI**
Intercept	0.017	-0.074	0.107
Location (north)	0.008	0.007	0.008
Log_{10} wing length	0.647	0.636	0.659
Age (juvenile)	0.007	0.006	0.007
Season (winter/dry)	-0.003	-0.003	-0.002
Year	1×10^{-5}	-1×10^{-5}	3×10^{-5}
Migration (nomadic)	0.044	-0.129	0.217
Migration (resident)	0.061	-0.117	0.238
Foraging method (visual)	-0.121	-0.317	0.078
**Model including location*migration interaction**			
**Random effects**	**Variance**		
Species	0.012		
Phylogeny	0.004		
Residual	0.001		
**Fixed effects**	**Estimate**	**Lower CI**	**Upper CI**
Intercept	0.016	-0.077	0.108
Location (north)	0.007	0.006	0.007
Migration (nomadic)	0.050	-0.124	0.225
Migration (resident)	0.065	-0.119	0.247
Log_{10} wing length	0.652	0.640	0.664
Age (juvenile)	0.007	0.006	0.007
Season (winter/dry)	-0.003	-0.003	-0.002
Year	1×10^{-5}	-1×10^{-5}	3×10^{-5}
Foraging method (visual)	-0.127	-0.325	0.073
Location (north) : migration (nomadic)	-1×10^{-4}	-0.002	0.002
Location (north) : migration (resident)	0.016	0.014	0.018
**Model including location*foraging interaction**			
**Random effects**	**Variance**		
Species	0.012		
Phylogeny	0.004		
Residual	0.001		
**Fixed effects**	**Estimate**	**Lower CI**	**Upper CI**
Intercept	0.015	-0.078	0.107
Location (north)	0.007	0.007	0.008
Foraging method (visual)	-0.128	-0.326	0.072
Log_{10} wing length	0.647	0.635	0.659
Age (juvenile)	0.007	0.006	0.007
Season (winter/dry)	-0.003	-0.003	-0.002
Year	1×10^{-5}	-1×10^{-5}	3×10^{-5}
Migration (nomadic)	0.051	-0.123	0.226
Migration (resident)	0.071	-0.112	0.254
Location (north) : foraging method (visual)	0.005	0.003	0.006

Table S3. Phylogenetic generalised linear mixed model output for predictors of absolute bill length of Australian shorebirds. Bill length was log-10 transformed to improve normality; differences in bill length shown are independent of differences in body size (i.e. without controlling for wing length, compare with Table S1 above). Effects are expressed relative to adult, migratory shorebirds in southern Australia, which use their bills to probe for prey, and were sampled during summer-spring or the wet season. Sample size is N = 99,443 observations from 30 shorebird species.

**Random effects**	**Variance**		
Species	0.024		
Phylogeny	0.011		
Residual	0.001		
**Fixed effects**	**Estimate**	**Lower CI**	**Upper CI**
Intercept	0.051	-0.079	0.182
Location (north)	0.005	0.004	0.005
Age (juvenile)	-0.001	-0.002	-0.001
Season (winter/dry)	-0.001	-0.001	-0.001
Year	2×10^{-5}	-2×10^{-6}	4×10^{-5}
Migration (nomadic)	0.037	-0.217	0.291
Migration (resident)	0.109	-0.152	0.368
Foraging method (visual)	-0.099	-0.381	0.181

Table S4. Phylogenetic generalised linear mixed model output for predictors of wing length of Australian shorebirds. Wing length was log-10 transformed to improve normality. Effects are expressed relative to adult, migratory shorebirds in southern Australia, sampled during summer-spring or the wet season. Sample size is N = 119,403 observations from 30 shorebird species. Model outputs show the model without interactions, referenced in the main results, and a model testing for an interaction between location and migration behaviour.

**Model showing main results**			
**Random effects**	**Variance**		
Species	0.009		
Phylogeny	0.003		
Residual	2×10^{-4}		
**Fixed effects**	**Estimate**	**Lower CI**	**Upper CI**
Intercept	0.077	0.023	0.130
Location (north)	-0.005	-0.005	-0.004
Age (juvenile)	-0.012	-0.013	-0.012
Season (winter/dry)	0.003	0.003	0.003
Year	-2×10^{-5}	-3×10^{-5}	1×10^{-5}
Migration (nomadic)	0.001	-0.148	0.150
Migration (resident)	0.085	-0.070	0.241
**Model including location*migration interaction**			
**Random effects**	**Variance**		
Species	3×10^{-4}		
Phylogeny	0.005		
Residual	2×10^{-4}		
**Fixed effects**	**Estimate**	**Lower CI**	**Upper CI**
Intercept	0.079	0.023	0.134
Location (north)	-0.004	-0.004	-0.004
Migration (nomadic)	-0.005	-0.161	0.151
Migration (resident)	0.083	-0.080	0.246
Age (juvenile)	-0.012	-0.013	-0.012
Season (winter/dry)	0.003	0.003	0.003
Year	-2×10^{-5}	-3×10^{-5}	1×10^{-5}
Location (north) : migration (nomadic)	5×10^{-4}	-5×10^{-4}	0.002
Location (north) : migration (resident)	-0.010	-0.011	-0.009

Table S5. Phylogenetic generalised linear mixed model output for predictors of body mass of Australian shorebirds. Mass was log-10 transformed to improve normality. Effects are expressed relative to adult, migratory shorebirds in southern Australia, sampled during summer-spring or the wet season. Sample size is N = 202,647 observations from 30 shorebird species. Model outputs show the model without interactions, referenced in the main results, and a model testing for an interaction between location and migration behaviour.

**Model showing main results**			
**Random effects**	**Variance**		
Species	0.043		
Phylogeny	0.061		
Residual	0.003		
**Fixed effects**	**Estimate**	**Lower CI**	**Upper CI**
Intercept	0.185	0.027	0.344
Location (north)	-0.030	-0.031	-0.029
Age (juvenile)	-0.052	-0.052	-0.051
Season (winter/dry)	0.046	0.046	0.047
Year	-1×10^{-4}	-1×10^{-4}	-1×10^{-4}
Migration (nomadic)	-0.008	-0.451	0.435
Migration (resident)	0.274	-0.194	0.741
**Model including location*migration interaction**			
**Random effects**	**Variance**		
Species	0.042		
Phylogeny	0.067		
Residual	0.003		
**Fixed effects**	**Estimate**	**Lower CI**	**Upper CI**
Intercept	0.186	0.031	0.343
Location (north)	-0.029	-0.030	-0.029
Migration (nomadic)	-0.013	-0.449	0.420
Migration (resident)	0.277	-0.184	0.733
Age (juvenile)	-0.051	-0.052	-0.051
Season (winter/dry)	0.046	0.045	0.046
Year	-1×10^{-4}	-1×10^{-4}	-1×10^{-4}
Location (north) : migration (nomadic)	0.002	-0.001	0.005
Location (north) : migration (resident)	-0.020	-0.024	-0.017

Table S6. Phylogenetic generalised linear mixed model output for predictors of body stores (i.e. mass controlling for wing length as an indicator of body size) of Australian shorebirds. Mass was log-10 transformed to improve normality. Effects are expressed relative to adult, migratory shorebirds in southern Australia, sampled during summer-spring or the wet season. Sample size is N = 118,017 observations from 30 shorebird species. Model outputs show the model without interactions, referenced in the main results, and a model testing for an interaction between location and migration behaviour.

**Model showing main results**			
**Random effects**	**Variance**		
Species	0.011		
Phylogeny	0.011		
Residual	0.003		
**Fixed effects**	**Estimate**	**Lower CI**	**Upper CI**
Intercept	0.028	-0.046	0.100
Location (north)	-0.028	-0.029	-0.027
Log_{10} wing length	1.752	1.732	1.772
Age (juvenile)	-0.026	-0.027	-0.025
Season (winter/dry)	0.037	0.036	0.038
Year	2×10^{-4}	2×10^{-4}	2×10^{-4}
Migration (nomadic)	-0.027	-0.231	0.180
Migration (resident)	0.111	-0.101	0.324
**Model including location*migration interaction**			
**Random effects**	**Variance**		
Species	0.011		
Phylogeny	0.011		
Residual	0.003		
**Fixed effects**	**Estimate**	**Lower CI**	**Upper CI**
Intercept	0.028	-0.046	0.101
Location (north)	-0.028	-0.029	-0.027
Migration (nomadic)	-0.032	-0.237	0.175
Migration (resident)	0.113	-0.100	0.326
Log_{10} wing length	1.750	1.730	1.770
Age (juvenile)	-0.026	-0.027	-0.026
Season (winter/dry)	0.037	0.036	0.038
Year	2×10^{-4}	2×10^{-4}	2×10^{-4}
Location (north) : migration (nomadic)	0.010	0.007	0.014
Location (north) : migration (resident)	-0.004	-0.008	-0.001

Supplementary Note 1: DATA CLEANING METHODS

Cleaning morphometric data

Community scientists of the VSWG and AWSG have expert knowledge of shorebird ecology and considerable experience in taking morphological measurements. Nevertheless, with so many observations of shorebirds taken over 45 years by numerous volunteers, it is inevitable that there will be occasional errors in the raw data. Hence, we cleaned our data prior to analysis to remove instances of clear errors. We removed errors for bill length, head-and-bill (HB) length, wing length and mass data while blind to sample location. HB length was not included in our analysis but was used to compare against bill length while cleaning the morphometric data. HB length is measured from the back of the skull to the tip of the bill using callipers. Firstly, we corrected measurements that appeared to be out by a factor of ten, for example, due to measurements recorded in centimetres instead of millimetres or because a decimal point was missed when transcribing the data. Secondly, we removed 'extreme' outliers (i.e. with values that were clearly impossible) from the entire dataset, irrespective of species: We excluded all bill lengths below two millimetres and above half a metre, HB lengths below 20 millimetres and above 0.8 of a metre, wing lengths below 50 millimetres and above 1.5 metres, and mass values below 5 grams and above two kilograms. For bill and HB lengths, we next used a multivariate outlier removal approach. Given the strong, predicted relationship between bill length and HB length for the same individual, we calculated the Mahalanobis distance from the estimated relationship between the two variables and removed points beyond the 'adjusted quartile' for the Mahalanobis distance (Filzmoser, 2004; Filzmoser et al., 2005). This approach enabled us to determine inaccurate values due to their departure from the expected relationship between the two variables. This includes errors for bill length, HB length, and where the combination of bill and HB lengths for the same individual gives unlikely values for head length, suggesting one or both measurements are inaccurate. The adjusted quartile is an adaptive indicator that attempts to distinguish between extreme values and true outliers based on departures from the predicted relationship (Filzmoser, 2004; Filzmoser et al., 2005). We calculated Mahalanobis distances and

adjusted quartiles using the package ‘mvoutlier’ in R (Filzmoser & Gschwandtner, 2018). We excluded likely errors for wing length and mass using set cut-offs for each species based on visual inspection of the data.

Data were initially recorded in datasheets, with ‘Datasheet ID’ assigned to a given species and capture event, where the same group of people typically completed individual datasheets. We therefore removed all data points for bill length, wing length and mass from datasheets with over 30% of measurements classed as outliers for bill length, wing length and mass respectively. We reasoned that if a sampling group made errors for at least 30% of their measurements, other measurements by the same group were likely inaccurate, even if they fell within the expected range of values.

We calculated repeatability of measurements taken from the same individuals following Nakagawa and Schielzeth (2010), dividing variance explained by individuals (band number) by the residual variance and individual variance combined. Variances were estimated for repeat captures using a linear mixed model with bill length, wing length or mass as the response variable, sample year, age (adult/juvenile) and season (summer-wet vs winter-dry) as fixed terms, and species and band ID as random effects. Repeatability for bill length = 0.92, and wing length = 0.72, for N = 3317 samples of 1583 individual shorebirds (band numbers) captured in the same year. Repeatability for mass = 0.78, for N = 3168 samples of 1567 individuals captured in the same month and year. Lower repeatability of wing length and mass are to be expected, as measurements are likely influenced by factors such as recent foraging success prior to capture and feather wear and tear. By comparison, bill lengths, though impacted by growth and wear, are expected to remain relatively consistent and this is likely reflected in the higher repeatability scores. Repeatability scores may underestimate true measurement accuracy due to possible errors in reading band numbers. We note that measurement error will most likely add noise to the dataset rather than systematic bias, because many of the same people collected shorebird data in both northern and southern Australia (the AWSG was founded by members of the VSWG), using consistent methods.

Assigning sex

Sex was initially estimated in the field based on a combination of plumage characteristics and sex differences in morphology for bar-tailed godwits, curlew sandpipers, far-eastern curlews, ruddy turnstones and sharp-tailed sandpipers. Taking advantage of the relatively substantial differences in the size and shape of males and females, we later estimated the sex of unsexed bar-tailed godwits and far eastern curlews, based on differences in bill length, and sharp-tailed sandpipers, based on differences in mass (Figure S6). This approach is consistent with previous shorebird research and necessary where molecular sexing methods are unfeasible (Higgins & Davies, 1996). However, we acknowledge such an approach is imperfect and likely to add noise to the dataset due to misclassified sexes. Nevertheless, including an estimate of sex in our analyses enables us to test for latitudinal patterns in morphology while controlling for possible effects of differential migration between the sexes in sexually dimorphic species (see Discussion in the main text). We did not use similar post-hoc methods to assign sexes to unsexed curlew sandpipers and ruddy turnstones due to the greater overlap of morphology between the sexes.

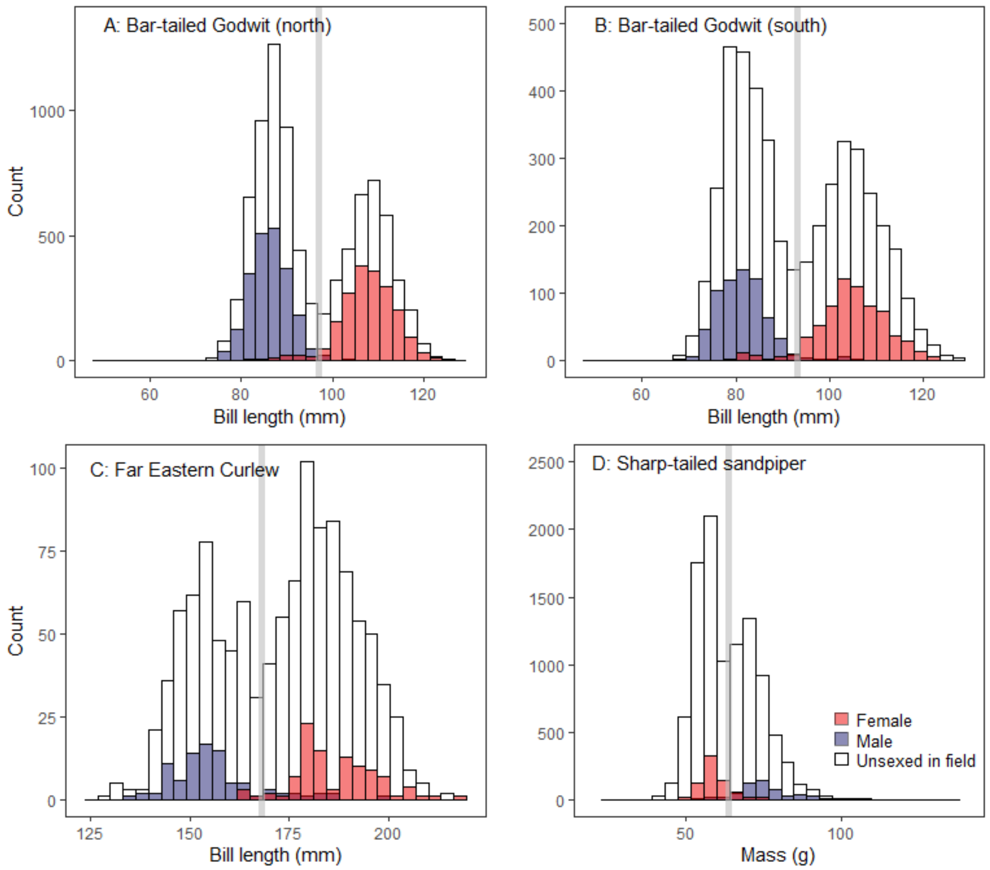

Figure S6. Bar plots showing cut-offs used to assign sexes to unsexed individuals (white bars) based on patterns from individuals initially sexed in the field (red = female; blue = male). Cut-offs are shown for the (A) northern Australian population of bar-tailed godwit (bill length > 97 mm = female), (B) southern Australian population of bar-tailed godwit (bill length > 93 mm = female), (C) far eastern curlew (bill length > 168 mm = female) and (D) sharp-tailed sandpiper (mass > 64 g = male). Separate cut-offs were used for northern and southern bar-tailed godwit populations due to consistent morphological differences between northern and southern Australian sub-species.

Supplementary References

Filzmoser, P. (2004). A multivariate outlier detection method. *Seventh International Conference on Computer Data Analysis and Modeling*, *1*(1989), 18–22. http://computerwranglers.com/com531/handouts/mahalanobis.pdf

Filzmoser, P., Garrett, R. G., & Reimann, C. (2005). Multivariate outlier detection in exploration geochemistry. *Computers and Geosciences*, *31*(5), 579–587. https://doi.org/10.1016/j.cageo.2004.11.013

Filzmoser, P., & Gschwandtner, M. (2018). *Package 'mvoutlier': multivariate outlier detection based on robust methods* (2.0.9). http://cstat.tuwien.ac.at/filz/ NeedsCompilation

Higgins, P. J., & Davies, S. J. J. F. (Eds.). (1996). *Handbook of Australian, New Zealand and Antarctic Birds. Volume 3: Snipe to Pigeons*. Oxford University Press.

Nakagawa, S., & Schielzeth, H. (2010). Repeatability for Gaussian and non-Gaussian data: A practical guide for biologists. *Biological Reviews*, *85*(4), 935–956. https://doi.org/10.1111/j.1469-185X.2010.00141.x

Supplementary Note 2: CONSORTIA MEMBERSHIPS

The following individuals are the members of the two consortia who have contributed to multiple field trips over the collection data period

Victorian Wader Study Group

Bev Abbott
Geoff Abbott
Rick Aitchison
Kahlid Al Dabbagh
Ruby Albury
Margaret Alcorn
Richard Alcorn
Heather Alexander
Charles Allen
Jocelyn Allen
Malcolm Allen
Terri Allen
Stephen Ambrose
Mark Anderson
Deborah Andrew
Peter Anton
George Appleby
Gabrielle Archard
Allen Archbold
Max Arney
Basil Artimedes
Bruce Atkin
Steve Atkinson
David Ball
Tony Ball
Alex Barisic
Ryan Barnaby
Lisa Barter
Mark Barter
Terry Barter
Kevin Bartram
Rose Baulch
Graham Beal
Jenny Beal
Lauren Beasley
Andy Bennett
Margaret Bennett
Mark Bennett
Gail Berry
Rob Berry
Mark Bezuijen
Robert Bezuijen
Steve Bianchi
Steven Bianchi
David Billinghurst
Pat Bingham
Margaret Blakers
Robin Borland
Janine Bossel
John Bowden-Perry
Adrian Boyle
John Brandenberger
Suzanne Brandenberger
Amanda Breidahl
Stephen Brend
Robert Brinkman
Judy Brown
Malcolm Brown
Prudence Brown
Andrew Browne
Sue Bryceson
Susanna Bryceson
Kate Buchanan
Anna Buchhorn
Paul Buchhorn
Alan Burbidge
Mavis Burgess
Nigel Burgess
Barbara Burns
Tanya Butcher
Hannah Buys
Bill Bygott
Ian Cairns
Dawn Cameron
Gordon Cameron
Margaret Cameron
Aiden Campbell
Jeff Campbell
Sarah Campbell
Thomas Cansse
Peter Carr
Catherine Cavallo
Jo Chadwick
Richard Chamberlain
Andrew Chapman
Ann Chappel
Anne Chappel
Mervyn Chappel
Christina Cheers
Kim Chen
June Cherrey
Smathie Chong
Alan Clarke

Allan Clarke
Allen Clarke
Rohan Clarke
Sue Clegg
Rob Clemens
Prue Clements
Bretan Clifford
David Close
Lisa Collins
Peter Collins
Charles Compton
Christine Connelly
Heather Connor
James Connor
Mike Connor
Robin Connor
Cecilia Cook
Elizabeth Cook
Marguerite Cordell
Julian Correia
Trish Corrie
Mary Cowling
Dave Cropley
Shirlene Cropley
Tahlia Cruise
Mark Cullen
Peter Curry
Monika Czastka
Wendy D'Amore
Amanda Dalgleish
Sue Danjko
Peter Dann
Steve Darby
Louise Dargaville
Rosemary Davidson
Stephen Davidson

Jeff Davis
Michael Dawkins
Bob Dawson
John Dawson
Geoff Deason
Norma DeGaris
Ren DeGaris
Julie Deleyev
Jill Dening
Xenia Dennett
Mary Dharmarajah
Meridith Dharmarajah
Vero Dharmarajah
Victoria Dharmarajah
Barbara Dickson
Jenna Diehl
Alexandra Djurovich
Michael Douglas
Joris Driessen
Lee Duclos
Graham Duell
James Dunlop
Andrew Dunn
Elizabeth Dunn
John Eckert
Dianne Emslie
Alice Ewing
John Fallan
Rebecca Fallan
Jon Fallaw
Erin Farmer
Susie Farquhar
David (Frankie) Farrar
Rory Ferguson
Alf Finch
Maureen Fitzgerald

Robin Fitzgerald
Jason Flesch
Mernie Foley
Melinda Ford
Berrice Forest
Amelia Formby
Richard Forster
Richard Forster
Charles Francis
Andrea Fullagar
Rob Gail
Batbayar Galbalt
Tim Gale
Brett Gardner
Stephen Garnett
Bev Geoff
Dave Gerard
Elizabeth Gerard
Angela Gibbs
Colin Gibbs
Gail Gibbs
Heather Gibbs
Melanie Gibbs
Peter Gibbs
Margie Gibson
Don Gillespie
Joyce Gillespie
Belinda Gillies
Dave Gillison
Kate Gorringe-Smith
Carlene Gosbell
Ken Gosbell
Andrew Gosden
Kath Gosden
Olivia Gourley
Kathryn Goyen

Doris Graham
Bob Green
Nathan Gregory
Nicole Grenfell
Patrick-Jean Guay
Jim Gunn
Surong Gunn
Tim Gunn
Jane Gurling
Julie Gurling
Susie Guthrie
Angie Gutowski
Tony Habraken
Paul Hackett
Elizabeth Haines
Nola Haines
Peter Hamilton
Graham Hancock
Petra Hanke
Birgita Hansen
Daniel Harley
Sue Harris
Susan Harris
Trish Hart
Neville Hatten
Robyn Hatten
Ruby Hatten
Tim Hatten
Heather Haughton
Peter Haughton
Peter Haward
Jenny Hawkins
Michael Hawkins
Becky Hayward
Brian Hayward
Toni Hayward
Ron Hedley
Barbara Henderson
David Henderson
Heather Hermans
Peter Hermans
Tony Hertog
Marilyn Hewish
Oki Hidayat
Faye Hill
Bindi Hillen
Jenny Hiscock
Trevor Hodson
Andrej Hohmann
David Hollands
Margaret Hollands
Patsy Holmen
Stewart Holohan
Jamie Holyoake
Vivien Holyoake
Yvonne Honey
Tracey-Ann Hooley
Peter Houston
Damian Howard
Bethany Hoye
Tania Ireton
Digger Jackson
Gavin Jackson
Don Jeans
Peter Jenkins
Angela Jessop
Suzanne John
Murray Johns
Penny Johns
Steve Johnson
Peter Johnstone
Eric Jones
Keith Jones
Silvia Jones
Peter Kasper
Daphne Keller
Ralph Keller
Matthew Kennedy
Greg Kerr
Barbara Khalifa
Debbie King
Steve Klose
Irma Kluger
Vivien Kluger
Joy Knight
Leona Knight
Femmie Kraaijeveld
Ken Kraaijeveld
Angus Lamin
Tessa Lamin
Geoff Lamour
Brett Lane
S.G. (Bill) Lane
Rowena Langston
Bruce Lavender
John Lawson
Rick Lebenhole
Casey Lee
Hannah Lee
Kit Lee
Daniel Lees
David Legg
Alan Leishman
Sally Leonard
John Lesku
Mark Lethlean
David Lewis
Jutta Leyrer

Shan Wei Rachel Liew
Amanda Lilleyman
Janet Limb
Simeon Lisovski
Laurie Living
Rodney Long
Moira Longden
Andy Longmore
Sue Longmore
Gabriel Low
Kim Lowe
Andrew Lowther
Sam Lowther
Debbie Loyn
Debby Loyn
Richard Loyn
Callum Luke
John Lyons
Susie Lyons
Donald MacMillan
Meg Macmillan
Grace Maglio
Bruce Male
Ben Malseed
Katrina Malseed
Kathryn Manago
Jan Mangan
Piper Mangan
Ila Marks
Brian Martin
John Martindale
Gary Matthews
Nigel Matthews
Golo Maurer
Bernie McCarrick
Ellen McCulloch
Clare McCutcheon
Geoff McDonald
Joan McDonald
Rodney McFarlane
John McKean
Mark McKinnon
Anne McMillan
Pat McWhirter
Krystii Melaine
Peter Melanie
Tove Melgaard
David Melville
Jan Merkel-Stol
Glynn Mihan
Eric Miller
Heidi Miller
Jannette Miller
Clive Minton
Ida Minton
Nigel Minton
Pat Minton
Roger Minton
Anthony Mitchell
Peter Mitchell
Stewart Monckton
Lorraine Moore
Chris Morris
Frank Morris
Helen Morris
Barbara Moss
Rowan Mott
Emma Moysey
Zebedee Isaac Muller
Melanie Mumford
John Munro
Lynn Murdoch
Brenda Murlis
Mick Murlis
Luke Naismith
Tracy Neilson
John Newman
Mike Newman
Bridget Nicholson
Philip Norman
Mathew Northward
Ghillies Nott
Jordan O'Neill
Maureen O'Neill
Paul O'Neill
Kim O'Riley
Rory O’Brien
Joanne Oldland
Ben Oliver
Geoff Osler
Juria Ozols
Joy Pagon
Michael Pagon
Priscilla Park
Graham Parkyn
Vicki Parkyn
Ian Pascoe
Penelope Pascoe
Linda Patrick
Rob Patrick
Robert Patterson
Reece Pedler
Simon Pender
Peter Penny
Petina Pert
Sara Petrovic
Hugo Phillipps
Jeremy Phillipps

Maria Phillipps
Alexander Phillips
Gareth Phillips
Roxanne Phillips
Shoreh Phillips
Gordon Phillips-Ross
David Phillipson
Heather Phillipson
Jim Phillipson
Alan Pilkington
Wendy Pilkington
Lois Platt
Warren Platt
Murray Portbury
Carolyn Pradun
John Pratt
Philip Pratt
Phillip Pratt
Mike Preeston
David Purchase
Steven Putt
Thomas Putt
Susan Quirk
David Rantall
Rebecca Rawlings
Marj Reni
Ann Renkin
John Renowden
April Reside
Jenny Reside
Jim Reside
Shane Reside
Laura Rhodes
Annabel Richards
Roger Richards
Bruce Ridgeway
Don Ripper
Jude Ripper
Alice Risely
Anthony Roberts
Bruce Robertson
David Robertson
Don Robertson
Greta Robertson
Minnie Robertson
Annie Rogers
Danny Rogers
Ken Rogers
Maryam Rogers
Joanne Rolland
Thierry Rolland
Jade Ronke
Natalia Rose
Paul Rose
Bob Ross
Diane Ross
Toby Ross
Oliver Rosznay
Margaret Rotherham
Yaara Rotman
Nancy Roussac
Neville Roussac
Matt Rowan
Chris Rowe
Graeme Rowe
Margaret Rowe
Michael Rowe (NSW)
Michael Rowe (VIC)
Luke Rozek
Megan Rush
Michelle Sabto
Sonia Sanchez Gomez
Liz Sarrailhe
Stuart Sarrailhe
Ira Savage
Debra Saxon-Campbell
Clinton Schipper
Tom Schmidt
Chris Scholz
Martin Schultz
Nicole Schumann
Samantha Setterfield
Kelly Shannon
Laurie Sharp
Cameron Sharpe
Julie Shaw
Kathy Shurcliff
Andrew Silcocks
Andrew Silcocks
Charles Silveira
Howard Simco
Holly Sitters
Jenny Skewes
Jenny Skewes
Rod Slater
Sue Slater
Alex Slavenko
Marta Slawuta
Alan Smith
Charles Smith
Colin Smith
Hannah Smith
Mark Smith
Mem Smith
Ron Smith
Shirley Smith
Anita Smyth
Bell Snow

Terry South
Vicki South
Roger Standen
Jon Starks
Philip Starks
Angela Steele
Bill Steele
Will Steele
Kieran Stephenson-Banks
Kate Stevens
Ian Stevenson
Jonathon Stevenson
Anna Stewart
Iain Stewart
James Stewart
Sally Stewart
Sandy Stewart
James Stockton
Tony Stokes
Annie Stoney
John Stoney
Julie Strudwick
Deb Sullivan
Alisa Swan
Bob Swindley
Robert Swindley
Sally Symonds
Naeko Takeuchi
Naoko Takeuchi
Laura Tan
Andrew Taylor
Christine Taylor
Jane Taylor
Susan Taylor

Deryn Thomas
Lynne Thomas
Claire Tingate
Amy Tipton
Pavel Tomkovich
Dale Tonkinson
Leon Trembath
David Trudgen
Wendy Trudgen
Lyn Turner
Lindsay Tyler
Stephanie Tyler
Megan Underwood
Jim Vadolas
John Vaitkunas
Connor Van Doorn
Gloria Van Duyne
Kay Van Loon
Paul Van Loon
Mary-Ann Van Trigt
David Van Veldhuisen
Helen Vaughan
Inka Veltheim
Cameron Villani
Joanne Walker
Mark Walker
Pam Walker
Ray Wallace
Caroline Walmsley
John Walmsley
Diane Walton
Nick Walton
Keith Ward
Doug Watkins

Hazel Watson
Marion Weaving
Will Webster
Dan Weller
Andrew Wells
Andrea West
Mike Weston
Norman Wettenhall
Anthea Whitelaw
Jim Whitelaw
David Wilbraham
Jean Wilbraham
Michelle Wille
Jennifer Williams
Ross Williamson
Kailash Willis
Annette Wills
David Wilson
Jeannine Wilson
Jim Wilson
Jack Winterbottom
Bob Winters
Diannne Winters
Jerry Wong
Peter Woodall
Sharon Woodend
Avron Woolf
Prue Wright
Dallas Wyatt
Jude Wyatt
Celine Yap
Bruce Yu
Meijuan Zhao
Elizabeth Znidersic

Australasian Wader Studies Group

Sue Abbotts
Nigel Adam
Joan Adams
John Allcock
Barbara Allen
Charles Allen
Terri Allen
Debbie Andrew
Rudy Anto
Claire Appleby
Alexandra Appleman
Alan Archbold
Howard Armstorng
Thais Armstrong
Zulfi Arsan
Jon Avon
Allan Baker
Bala Balachandran
Paul Barden
John Bardsley
Yahkat Barshep
Cam Bates
Chris Bell
Terry Bell
Keith Bellchambers
Maria Bello
Loyd Berry
Rob Berry
Demetrios Bertzeletos
Rachel Blakey
Adrian Boyle
Nicholas Branson
Dave Bridges
Bob Brinkman
Judy Brown
Malcolm Brown
Rebucca Brugman
Bee (Birgitta) Buche
Simon Buckell
Anna Buckhorn
Paul Buckhorn
Robert Bush
Andrew Cadman
Rainy Cai
Jeff Campbell
Michael Campbell
Sarah Campbell
Peter Challinor
Ginny Chan
Simba Chan
Ray Chatto
Andiball Chen
Chih-Hao Chen
Fion Cheung
Chung-Yu Chiang
Eric Ching-Chih
Rebekah Christensen
Igor Chupin
Alan Clarke
Allan Clarke
Tom Clarke
Bretan Clifford
Jon Coleman
Peter Collins
Jesse Conklin
Ian Connell
Graham Couchman
Phil Craven
Peter Crighton
Tony Crocker
Patricia Croft
Ruth Croger
Vicki Cronan
Mike Crone
Dave Cropley
Mike Crowley
Candy Curran
Alastair Cuthbert
Anne Cuthbert
Lucy Dadour
Delip Das
Chris Davey
Rosemary Davidson
Simon Davies
Alison Dawes
Mike Dawkins
Sarah Dawkins
Peter de Boer
Petra de Goej
Loes de Jong
Nicki de Prue
Tom Delaney
Julie Deleyev
Xenia Dennett
Roy Dennis
Belinda Dettmann
Mandy Dey
Alison Dick
Jamie Dick
William Dick
Barbara Dickson
Leon Dikstra
Petra Dikstra
Jing Jing Ding
Steve Dodd

Mark Dodds
David Douglas
Tegan Douglas
Penny Drake-Brockman
Joris Driessen
David Drynan
Richard du Feu
Andre Duiven
James Dunlop
Jodie Dunn
Kathryn Dyball
Leander Dykstra
John Eckert
Dennis Elphick
Rick Else
Glen Emkie
Dianne Emslie
Josh Engel
Brian Etheridge
Simon Etheridge
Lee Eun Soo
Alice Ewing
Toby Fagerstrom
Qian Fawan
Iwan "Londo" Febrianto
Meng-Chie Feng
Bruce Ferry
Mark Field
Don Finch
Louise Finch
Kathy Fletcher
Milly Formby
Simon Foster
Diane Fraser
Don Fraser
Pat French
Joan Fried
Graham Fry
Peter Fullagar
Daphne Fullager
Ash Furneax
James Gan
Rob Ganly
John Geale
Les George
David Gerard
Yuri Gerasimov
Colin Gibbs
Dominic Gibbs
Gail Gibbs
Heather Gibbs
Betty Gilbert
Victor Glupov
Patricia Gonzalez
Carlene Gosbell
Ken Gosbell
David Goulding
Olivia Gourley
Doris Graham
Gordon Graham
Keith Grant
Mark Grantham
Ros Green
Nathan Gregory
Zang Guangming
Patrick Guay
Tim Gunn
Chu Guozhong
Daniel Gustafsson
Angie Gutowski
Kerry Hadley
Hugh Hanmer
David Han-Po Chang
Birgita Hansen
Joyce Harding
Sandra Harding
Anne Hardman
Joe Hardman
Daren Harris
Ken Harris
Heather Haughton
Peter Haughton
Robina Haynes
Peng He
Jacquie Heaton
Nicola Hedges
Klaus Hein
Henke Heinekamp
Andrew Hemsley
Barbara Henry
Dennis Henry
Josie Hewitt
Richard Holmes
Vivien Holyoake
Zang (Rick) Hongxi
Jiang Hongxing
Amy Huang
Falk Huettmann
Kuan-Chieh Hung
James Hutchinson
Liz Huxtable
Gemma Insley
Hugh Insley
Phil Ireland
Digger Jackson
Gavin Jackson
Micha Jackson
Don Jeans

Peter Jenkins
Angela Jessop
Xin Jin
Tony John
Steve Johnson
John Jones
Phil Joy
Hirotaka Kantani
Minoru Kashiwagi
Sharon Kast
Antti Kause
Mianjuan Ke
Steve Kendall
James Kennerley
Greg Kerr
Nicole Kerr
Justine Keuning
Lindall Kidd
Han-Byal Kim
Debbie King
Phil Kingston
Hirotaka Kitani
Wally Klau
Margrit Klein
Steve Klose
Irma Kluger
Kristen Knight
Eva Kok
Ramakrishnan Kolandavelu
Shigemoto Komeda
Christian Kouroumihalis
Jan Kube
Murray Kun-Chang Li
Martin Kunze
Emilia Lai
Ken Lance
Brett Lane
Rowena Langston
Elana Lappo
Keith Larsen
Anna Lashko
Arnaud Laudelot
Sam Lawrence
Eila Lawton
Adam Leavesley
Roger Lee
Alan Leishman
Ning Le-Ning Chang
Sally Leonard
Katherine Leung
Jan Lewis
Jenny Lewis
Jutta Leyrer
David Li
Jing Li
Ma Li
Tz-Yu Liao
Amanda Lilleyman
Haw Chuan Lim
Charlene Lin
Hung-Ju Lin
Mu-Ming Lin
Geoff Linkleter
Simeon Lisovski
Brian Little
Greg Little
Judy Little
Wei-ting Liu
Mike Lofthouse
Wendy Lofthouse
Moira Longdon
Samantha Lostrom
Richard Loyn
Helen Macarthur
Rosemary Macarthur
Meme Macdonald
Mark MacFadden
Meg MacMillan
Trixi Maddon
Peter Madvig
Kajonori Maehara
Grace Maglio
Joyce Magor
Bruce Male
Jan Mangan
Tohru Manu
Ila Marks
Kevin Marshall
John Martindale
Tashiyuki Matsumura
Takeyoshi Matsuo
Gary Matthews
Andrew McCreery
Jim McGuiness
Leonie McMahon
Pat McWhirter
David Melville
Julia Melville
Glynn Mihan
Nigel Milius
Eric Miller
Heidi Miller
Ken Mills
Ruth Mills
David Milton
Christopher Minton
Clive Minton
Nicholas Minton

Nigel Minton
Pat Minton
Roger Minton
Amare Mitamura
Kimiko Mohri
Vladimir Morozov
Nadezda Morozova
Amdrew Moss
Dorian Moss
Gillian Moss
Martin Moss
Otto Mueller
Tadao Muira
Lys Muirhead
Gabriele Muller
Taej Mundkur
John Munro
Brenda Murlis
Mick Murlis
Barbara Myers
Jeong-Yeon Myung-Suk
Noboru Nakamura
Bruce Neimann
Peter Newberry
Larry Niles
Somchai Nimnuan
Misao Nishimra
Mas Noerdjito
Heidi Nore
Richard Norman
Ian Northcott
Frank O'Connor
Maurice O'Connor
Takeshi Ogura
Takashi Okumura
Dave Oldham
Pat Oldham
Kim Onton
Jake Owens
Kikyoaki Ozaki
Noai Ozaki
Duncan Parish
Jin-Young Park
Graeme Parkyn
Ian Paterson
Rob Patrick
Rattapan Pattararangsan
Iain Patterson
Martina Patterson
Vaughan Patterson
Grant Pearson
Mark Peck
Reece Pedler
Hannes Pehlak
Hebo Peng
Ali Pentalow
David Pentalow
Petina Pert
Ilse Pickerd
Anne Pienkowski
Mike Pienkowski
Theunis Piersma
Sioux Plowman
Anne Preston
David Price
Elizabeth Price
Ma Qiang
Ji Qui
Bai Quiquan
Wendy Radford
Kelly Rainer
Chelsea Ralls
Kelly Rayner
Judith Read
Magda Remisiewicz
Marj Reni
John Renowden
Kathryn Renowden
April Reside
Zoya Reviakina
Steve Reynolds
Sue Rice
Annabel Richards
Brooke Richards
Roger Richards
Allan Richardson
Adrian Riegen
Henry Robb
Antony Roberts
Vivienne Roberts
Billy Robin
Kate Robin
Libby Robin
Tom Robin
Rob Robinson
Annie Rogers
Danny Rogers
Ken Rogers
Liz Rosenberg
Jill Rowbottom
Chris Rowe
Graham Rowe
Claudia Ruby
Mavis Russell
Beck Ryan
Alex Sapoznikov
Ira Savage
Andrea Schafer

Frances Schmeckel
Francoise Schmidt
Axel Schmidt
Jonny Schoenjohn
Rob Schuckard
David Seay
Betty Seddon
Kawaguchi Sentaro
Hila Shamir
Ian Shann
Simon Shih Han
Jarunee Siengsanan
Andrew Silcocks
Howard Simco
Holly Sitters
Humphrey Sitters
Ederic Slater
Matt Slaymaker
Annabel Slettenhaar
Jenny Smart
Mark Smart
Harry Smit
Hannah Smith
Roy Smith
Janet Sparrow
Brian Speechley
Robyn Spencer
David Spreadbury
Roger Standen
Claire Stevenson
Anna Stewart
Iain Stewart
James Stewart
Sally Stewart
Sandy Stewart
Susie Stockwell

Rob Stone
Eugeny Strekrikov
Vera Struenson
Ed Stubbing
Dieter Stürmer
George Swan
Bob Swindley
Barbara Swinfen
Roger Swinfen
Sally Symonds
Eugene Syroechovski
Yukio Takeda
Masabumi Takeshita
Naoko Takeuchi
Kun Tan
Susan Taylor
Bobbie Telford
Brian Thompson
Clare Thomson
LeDinh Thuy
Chiao (Judy) Ting
Allen To
Pavel Tonkovich
Tony Tree
Declan Troy
Chia-yang Tsai
Augustine Tuuga
Yasuo Ueki
Loes van den Bremer
Joop van Eerbeek
Hoang van Thang
Mary-Ann van Trigt
Gillian Vaughan
Mary Vaughan
Dick Veitch
Inka Veltheim

Petra Verburg
Mo Verhoeven
Yvonne Verkuil
Diane Walton
Mitchell Walton
Natalie Walton
Nick Walton
Rachel Walton
Junyan Wang
Lorine Wang
Xaun Wang
Sawai Wanghona
Lim Wanho
Joan Ward
Nik Ward
Robin Ward
Barbara Warren
Alistair Watson
Daphnie Watson
Hazel Watson
Katherine Watson
Mike Watson
Toni Webster
Wu Wei
Jing Weiguo
Bryce Wells
Gale Wells
Xue Wenjie
Mike Weston
Norman Wettenhall
Nell White
David Wilbraham
Jean Wilbraham
Carole Williams
Craig Williams
David Williams

Anne-Marie Wilson
Jim Wilson
Rob Wilson
Jamie Wood
Sharon Woodend
Prue Wright
Prue Wright
Wang Xiaofei
Feng Xuesong
Jeong-Yeon Yi
Chan Ying Chi
Erina Young
Gao Yu-ren
Alexander Yurlov
Sabine Zelz
Kejia Zhang
Shoudong Zhang
Xuan (Brenda) Zhang
Ma Zhijun
Qianyan Zhou
Bingrun Zhu
Liz Znidersic
Vladimir Zykov
