# Supplementary material for: Thermal adaptation best explains Bergmann’s and Allen’s Rules across ecologically diverse shorebirds
Source: Nat Commun. 2022 Aug 11;13:4727. doi: 10.1038/s41467-022-32108-3 (PMC9372053; doi:10.1038/s41467-022-32108-3)
Supplement: Supplementary file 3 — Reporting Summary [file 41467_2022_32108_MOESM3_ESM.pdf]

nature portfolio

Corresponding author(s): Matthew Symonds

Last updated by author(s): Jun 17, 2022

# Reporting Summary

Nature Portfolio wishes to improve the reproducibility of the work that we publish. This form provides structure for consistency and transparency in reporting. For further information on Nature Portfolio policies, see our Editorial Policies and the Editorial Policy Checklist.

## Statistics

For all statistical analyses, confirm that the following items are present in the figure legend, table legend, main text, or Methods section.

| n/a | Confirmed | |
|---|---|---|
| ☐ | ☒ | The exact sample size (*n*) for each experimental group/condition, given as a discrete number and unit of measurement |
| ☐ | ☒ | A statement on whether measurements were taken from distinct samples or whether the same sample was measured repeatedly |
| ☐ | ☒ | The statistical test(s) used AND whether they are one- or two-sided<br>*Only common tests should be described solely by name; describe more complex techniques in the Methods section.* |
| ☐ | ☒ | A description of all covariates tested |
| ☐ | ☒ | A description of any assumptions or corrections, such as tests of normality and adjustment for multiple comparisons |
| ☐ | ☒ | A full description of the statistical parameters including central tendency (e.g. means) or other basic estimates (e.g. regression coefficient) AND variation (e.g. standard deviation) or associated estimates of uncertainty (e.g. confidence intervals) |
| ☐ | ☒ | For null hypothesis testing, the test statistic (e.g. *F*, *t*, *r*) with confidence intervals, effect sizes, degrees of freedom and *P* value noted<br>*Give P values as exact values whenever suitable.* |
| ☐ | ☒ | For Bayesian analysis, information on the choice of priors and Markov chain Monte Carlo settings |
| ☒ | ☐ | For hierarchical and complex designs, identification of the appropriate level for tests and full reporting of outcomes |
| ☐ | ☒ | Estimates of effect sizes (e.g. Cohen's *d*, Pearson's *r*), indicating how they were calculated |

*Our web collection on statistics for biologists contains articles on many of the points above.*

## Software and code

Policy information about availability of computer code

| | |
|---|---|
| Data collection | No specialized software was used for data collection |
| Data analysis | All data analyses were run using R (version 4.0.2) and existing R packages (with version numbers) that have been published in the literature, as described in the text. |

For manuscripts utilizing custom algorithms or software that are central to the research but not yet described in published literature, software must be made available to editors and reviewers. We strongly encourage code deposition in a community repository (e.g. GitHub). See the Nature Portfolio guidelines for submitting code & software for further information.

## Data

Policy information about availability of data

All manuscripts must include a data availability statement. This statement should provide the following information, where applicable:

- Accession codes, unique identifiers, or web links for publicly available datasets
- A description of any restrictions on data availability
- For clinical datasets or third party data, please ensure that the statement adheres to our policy

The data we used in this study are freely available to download from the Dryad data repository and can be accessed with DOI link: https://doi.org/10.5061/dryad.xsj3tx9j5

# Field-specific reporting

Please select the one below that is the best fit for your research. If you are not sure, read the appropriate sections before making your selection.

- [ ] Life sciences
- [ ] Behavioural & social sciences
- [x] Ecological, evolutionary & environmental sciences

For a reference copy of the document with all sections, see nature.com/documents/nr-reporting-summary-flat.pdf

# Ecological, evolutionary & environmental sciences study design

All studies must disclose on these points even when the disclosure is negative.

| | |
|---|---|
| Study description | We compare the size (body mass and wing length) and bill length of shorebirds in tropical, northern Australia with members of the same species in temperate southern Australia. The analysis is repeated across 30 different species. We show that shorebirds follow Allen's and Bergmann's Rules and make inferences of the possible drivers of these rules based on the ecology of the different shorebird species in our study. |
| Research sample | The data consist of existing morphometric measurements made by members of the Victorian Wader Study Group and Australasian Wader Studies Group of individual birds from 30 Australian shorebird species that were caught, banded (and subsequently released) at locations in the north-west and south-east of Australia over a period of 46 years (1975-2021). Data include bill and wing length, and body mass measurements from shorebirds, as well as a description of wing moult, age and for some species, sex. Sample sizes for our main analyses are N = 99,443, 119,403 and 202,647 individuals for bill length, wing length and body mass respectively. |
| Sampling strategy | Where possible we included data on all individual shorebirds that have ever been recorded by the VWSG and AWSG since 1975. Unreliable records were excluded (see data exclusion below). For the analysis, we included only species that had at least N = 20 samples for bill, wing and mass measurements in both northern and southern Australia (the two regions compared in our analysis). We reasoned a sample of 20 was the minimum sufficient to give a reasonable estimate of species morphology in both regions (accounting for possible measurement errors etc). This criteria was determined prior to analysis and all species that met this criteria were included in the analysis. The significant effect observed the smallest sample comparison (bill length for 20 individuals from northern Australia for the greater sand plover) indicates post-hoc that this sampling threshold is sufficient to pick up biological effects. |
| Data collection | Data on morphometric measures were collected by members of the Victorian Wader Study Group and Australasian Wader Studies Group over the period (1975-2021). Members of the VWSG and AWSG typically caught shorebirds using cannon-nets while the birds were roosting during high tide. Bill length was measured as the exposed culmen (tip of bill to base of feathers) to the nearest 0.1 mm using callipers. Wing (maximum chord) length was measured while straightened and flattened using a butt-ended ruler, from the shoulder to tip of the longest primary feather, to the nearest 1 mm (larger species) or 0.1 mm (smaller species). Wing length measurements were excluded for birds moulting their ninth or tenth primary wing feather. Birds were weighed using scales to determine body mass to the nearest 1 g (larger species) or 0.1 g (smaller species). All individuals were banded and hence individual ID was noted, along with age (based on feather wear) and, where possible, sex (based on plumage dimorphism characteristics). Data were recorded manually by hand using pencil on paper before later being transferred to computer. We also collected data on the migration and foraging behaviour for the shorebird species included in our study from the Handbook of Australian, New Zealand and Antarctic Birds (‘HANZAB’; Marchant & Higgins 1993; Higgins & Davies 1996)). |
| Timing and spatial scale | We used an existing dataset collected by citizen scientists who have been monitoring shorebird populations and studying their natural history since 1975. The data used in our analysis includes measurements collected every year from 1975 to 2021. Data were collected in north-western Australia (close to Broome, Western Australia) and south-eastern Australia (coastal areas of South Australia, Victoria and Tasmania). |
| Data exclusions | Working with the existing dataset: we included all species with at least 20 measurements for bill length, wing length and body mass in both northern and southern Australia (the two regions compared for our analysis). We cleaned morphological data of expected errors prior to analysis using set, objective criteria (described in detail in the supplementary material): We excluded outliers for bill length based on the expected relationship between bill length and head-and-bill length (removing data points beyond the adjusted quartile for the Mahalanobis distance). We removed likely errors for wing length and body mass using predetermined cut-offs for each species based on our visual inspection of the data. Data were initially recorded on datasheets in the field, with a 'Datasheet ID' assigned to each; we removed all data for bill length, wing length and mass from datasheets with a high proportion of likely errors determined by the criteria above (> 30% 'likely errors'). We reasoned that if the sampling group made errors for at least 30% of the measurements, other measurements by the same group were likely inaccurate, even if they fell within the expected range of values. We excluded wing length measurements from birds that were moulting their primary wing feathers. |
| Reproducibility | Our study is observational only, based on morphological data collected in the field, and therefore does not contain reproducible experiments. |
| Randomization | As our study is observational, we did not assign subjects to randomized groups/treatements. |
| Blinding | As data were collected in the field at known field sites over 46 years, it was not possible for observers to have been blind to the locality of the morphological measurements. However, the observers would not have known of the use of the data, or subsequent hypothesis testing, presented in this study. The morphological data used in our analysis were cleaned of expected errors prior to analyses and while blind to the key variables we were testing (including sample location). |

Did the study involve field work? [ ] Yes [x] No

# Reporting for specific materials, systems and methods

We require information from authors about some types of materials, experimental systems and methods used in many studies. Here, indicate whether each material, system or method listed is relevant to your study. If you are not sure if a list item applies to your research, read the appropriate section before selecting a response.

## Materials & experimental systems

| n/a | Involved in the study |
|---|---|
| ☒ | ☐ Antibodies |
| ☒ | ☐ Eukaryotic cell lines |
| ☒ | ☐ Palaeontology and archaeology |
| ☒ | ☐ Animals and other organisms |
| ☒ | ☐ Human research participants |
| ☒ | ☐ Clinical data |
| ☒ | ☐ Dual use research of concern |

## Methods

| n/a | Involved in the study |
|---|---|
| ☒ | ☐ ChIP-seq |
| ☒ | ☐ Flow cytometry |
| ☒ | ☐ MRI-based neuroimaging |
